# A bacterial and viral genome catalogue from Atlantic salmon highlights diverse gut microbiome compositions at pre- and post-smolt life stages

**DOI:** 10.1186/s42523-025-00453-5

**Published:** 2025-08-11

**Authors:** Varsha Kale, Germana Baldi, Martin Beracochea, Cecilie Clausen, Alejandra Escobar-Zepeda, Sabina Leanti La Rosa, Laurène A. Lecaudey, Sen Li, Sarah S. T. Mak, Michael D. Martin, Garazi Martin Bideguren, Louisa A. Pless, Jacob A. Rasmussen, Alexander B. Rogers, Harald Sveier, Arturo Vera-Ponce de León, Ana Verissimo, M. Thomas P. Gilbert, Lorna Richardson, Morten T. Limborg, Robert D. Finn

**Affiliations:** 1 European Molecular Biology Laboratory, European Bioinformatics Institute (EMBL-EBI), Wellcome Genome Campus, Hinxton, Cambridge, UK; 2 Center for Evolutionary Hologenomics, Globe Institute, University of Copenhagen, Copenhagen, Denmark; 3 Department of Natural History, NTNU University Museum, Norwegian University of Science and Technology (NTNU), Trondheim, Norway; 4 Lerøy Seafood Group ASA, Bergen, 5020 Norway; 5 Faculty of Chemistry, Biotechnology and Food Science, Norwegian University of Life Sciences, Ås, Norway; 6 Faculty of Biosciences, Norwegian University of Life Sciences, Ås, Norway

## Abstract

Resolving the microbiome of the Atlantic salmon *Salmo salar* gut is challenged by a low microbial diversity often dominated by one or two species of bacteria, and high levels of host contamination in sequencing data. Nevertheless, existing metabarcoding and metagenomic studies consistently resolve a putative beneficial *Mycoplasma* species as the most abundant organism in gut samples. The remaining microbiome is heavily influenced by factors such as developmental stage and water salinity. We profiled the salmon gut microbiome across 540 salmon samples in differing conditions with a view to capture the genomic diversity that can be resolved from the salmon gut. The salmon were exposed to 3 different nutritional additives: seaweed, blue mussel protein and silaged blue mussel protein, including both pre-smolts (30-60 g salmon reared in freshwater) as well as post-smolts (300–600 g salmon reared in saltwater). Using genome-resolved metagenomics, we generated a catalogue of 11 species-level bacterial MAGs from 188 input metagenome assembled genomes, with 5 species not found in other catalogues. This highlights that our understanding of salmon gut microbial diversity is still incomplete. A prevalent bacterial genome annotated as *Mycoplasmoidaceae* is present in adult fish, and a comparison of functions revealed significant sub-species variation. Juvenile fish have a different microbial diversity, dominated by a species of *Pseudomonas aeruginosa*. We also present the first viral catalogue for salmon including prophage sequences which can be linked to the bacterial MAGs.

## Introduction

Aquaculture food production has witnessed a dramatic growth in the last 20 years, to ensure supplies of consumable fish meet the growing demands [1]. Concomitantly, the use of wild fish in aquaculture feed has been reduced, resulting in a need to find sustainable feed alternatives to fish meal, such as increasing the percentage of plant-based ingredients in the feed, and utilising novel additives from extractive species that obtain nutrients and filter waste from the surrounding environment. Extractive species are typically found in the same environment as the farmed fish, e.g. seaweed and molluscs. Patterns in the fish gastrointestinal microbiome have been scrutinised in response to the new feeds, as have phenotypic traits such as fish growth, immunity and feed conversion [1]. However most aquaculture-associated microbiome studies have employed a metabarcoding approach (using the small subunit (SSU) ribosomal RNA (rRNA) gene), to study fish species [2–8] including those that are closely related to Atlantic salmon (*Salmo salar*), such as Atlantic cod (*Gadus morhua*) and rainbow trout (*Oncorhynchus mykiss*) [9–11]. Metabarcoding approaches only provide taxonomic profiles, and the resolution of closely-related organisms to a species level is difficult to obtain [12].

Meanwhile, microbiome research has witnessed a transition towards genome-resolved analysis due to the development of advanced assembly and binning tools allowing taxa-specific prokaryotic and eukaryotic metagenome assembled genomes (MAGs) to be derived from shotgun sequencing data. Often these MAGs are aggregated into catalogues representing the taxonomic composition of diverse biomes including animal associated-gut environments [13, 14]. While genome-resolved analyses for the salmon gut microbiome are limited to a small number of studies, Rasmussen et al. [15] and Cheaib et al. [16] each resolved a single MAG in the genus *Mycoplasma*, and Rasmussen et al. [17] assembled a small catalogue of 19 MAGs. Vera-Ponce de León et al. [54] on the other hand, reported a much larger catalogue of 211 bacterial genomes comprising long-read-based isolate genomes and short-read-based MAGs. While each study included a *Mycoplasma* genome, those genomes represented different individual species in each case. Rasmussen et al. [15] compared their single *Mycoplasma* MAG to other host-associated *Mycoplasma* species to define its phylogenetic placement. However, this comparison was made at a species level, leaving any sub-species variation of this essential salmon symbiont, yet to be explored.

Whilst *Mycoplasma* genomes have been reported to dominate in existing salmon gut microbiome studies [8, 15–19], the microbiome of the fish gastrointestinal tract is heavily influenced by a range of environmental factors. The anadromous life cycle of salmon necessitates growth phases in both freshwater and marine water due to smoltification - the migration of juvenile fish from freshwater to seawater, and these diverging environments have been identified as an important factor shaping overall microbiota composition in fish [20, 21]. In addition, the microbial diversity in fish-gut varies during development, and undergoes a compositional change from juvenile pre-smolt to adult fish [8].

Looking beyond the cellular microbial composition of the fish-gut microbiome, viruses, considered the most abundant biological entity in the marine environment [22, 23], are both causative agents of many fish diseases, and reservoirs for potential future therapies (i.e phage therapy) [24]. Phages are key modulators of gut microbiomes and phage predation has a direct impact on infected bacteria as well as indirect effects such as disrupting microbial composition resulting in alterations in the gut metabolism [25–28].

The HoloFood project is a Horizon 2020 Research and Innovation Action funded project, which conducted a study of Atlantic salmon gut samples with the aim to characterise the microbiome in response to novel sustainable feeds. The fish samples included both pre- and post- smolt life stages, grown in freshwater and saltwater environments, and administered feed additives: blue mussel or seaweed. This study was conducted in controlled environments enabling the comparison of microbial species abundance in fish exposed to different feed, salinity and growth development stages. We present a high-quality MAG catalogue of 11 bacterial species and compare the HoloFood MAG catalogue to existing public MAGs to demonstrate that the HoloFood dataset contributes new species to existing salmon gut metagenome resources. Given the prevalence and abundance of *Mycoplasmoidaceae* species, we use methods to explore sub-species variation. We also explored the samples’ viral diversity, and detected other potential mobile genetic elements that may give rise to microbial genome plasticity in the relatively simple community found in the salmon gut. Thus we also present the first viral and plasmid catalogues for a fish-gut metagenome.

Genome-resolved metagenomics pertains to detailed analysis of functions, and consequently an estimation of sub-species, which is a level of resolution that cannot be achieved through metabarcoding alone. Salmon gut microbiome studies are limited to metabarcoding approaches, partly due to high levels of host contamination in shotgun metagenomic samples of gut content [9, 29]. This study highlights that we can successfully resolve genomes from the salmon gut microbiome, by sampling from a range of conditions such as: feed additive and developmental stage using a combined single run and co-assembly approach. We consistently identify novel species not found previously in the limited number of existing genome-resolved fish gut studies, demonstrating that the microbiome of the salmon gut is not yet saturated, but limited by host contamination and dominant species.

## Methods

### Source of sample data

By combining samples across multiple growth trials we built a novel and qualitative microbial gut catalogue for farmed Atlantic salmon across numerous relevant variables. Samples were taken from three trials in a recirculating aquaculture system (RAS), encompassing both juvenile and adult life stages, varying salinity conditions and novel sustainable feed additives: trial A - fermented algae (post-smolts in saltwater (22 psu)), trial B - blue mussel protein (post-smolts in saltwater) and trial C - silaged blue mussel protein (pre-smolts in freshwater). Samples from trial A and B were taken at day 0 (prior to the start of the trial) and at the end of the trial on day 60. Samples from trial C were taken at day 0 and at the end of the trial on day 77. At the start of each trial we sampled 60 individuals, and at the end of each trial we sampled 60 individuals from the control group and 60 from the test group with maximum inclusion of either seaweed (Trial A) or blue mussel protein (Trials B and C). This setup resulted in gut content samples from 180 salmon per trial and a total of 540 samples for characterising the salmon gut metagenome via shotgun sequencing. See Additional file 4 for more details on the background and rationale for the experimental design of the trials.

At each sampling event, fish were carefully sedated before sampling following all ethical requirements for EU standards, using an overdose of Finquel${\circledR }$ (Tricaine Methanesulfonate, MS-222). From each fish we sampled the intestinal gut content for shotgun-based metagenomics profiling. Samples from the distal gut content of the 60 individuals underwent DNA extraction and purification following the methods of Bozzi et al. [4]. Extracted DNA was quantified, and between 300 to 400 ng of DNA was sheared to a mean-length of 400 bp. For trials A and B sequencing library preparation was performed on each sample using the BEST single-tube protocol [30] and custom blunt-end adapters [31]. A unique index was incorporated into each library using custom-indexed primers during the indexing PCR. The optimal number of PCR cycles was individually estimated for each library using an Mx3005 qPCR machine (Agilent Technologies). The amplified libraries were then purified, pooled in equal molarity and sequenced by BGI-DK using 150 PE chemistry on the MGISeq 2000 platform. For Trial C DNA was extracted similarly to Trials A and B samples, but hereafter the DNA was shipped to Novogene (Cambridge, UK) for following QC, library building and Illumina sequencing using the Novaseq PE 150 platform. Since the primary aim of this paper is to best characterise the metagenomics reads in all trials, rather than make comparisons across the three trials, the use of a different sequencing platform for trial C samples is not expected to impact our conclusions. Throughout DNA extraction and library preparation six negative controls per 96-well plate were included to detect DNA from putative contaminant microbes. The concentration of each control sample was measured at different steps of the library preparation. There was no detectable DNA in the negative control samples, therefore we can conclude that our data were not affected by contamination during DNA extraction and library preparation. Full protocols for extraction, library preparation and sequencing of HoloFood salmon samples are detailed in [32].

### MAG generation

Shotgun metagenomic sequencing reads from 538 salmon gut samples were subjected to a standardised quality control workflow. Adapter and sequence trimming was performed with the AdapterRemoval [33] tool, trimming bases with a quality score <30, reads with >5 ambiguous bases, and finally reads with length <100 bp. Duplicate reads were discarded using SeqKit rmdup [34]. The remaining reads were mapped to the salmon host reference genome with bwa-mem2, and samtools [35, 36] was used to exclude sequences matching to the host salmon reference genome, GCA_905237065.2. The reads passing quality control were assembled per individual run using metaSPAdes v3.14.1 [37]. Subsequently, co-assemblies were performed to identify any additional microbial diversity not previously recovered. This included a co-assembly of samples from trial A and B separately, and co-assemblies of all samples from both trials. In light of the size of the dataset, MEGAHIT [38] was used to perform the co-assemblies due to its lower memory footprint. Trial C runs were not co-assembled together with A and B as the experimental design was different - the fish were juvenile pre-smolts farmed in freshwater and assembled separately due to the expected difference in presence of key taxa. The resulting assembled contigs were subjected to further quality control and filtering, namely the removal of any contigs coming from salmon or PhiX and the removal of contigs <500bp in length.

These resulting assemblies were then binned into genomes using MetaWRAP [39], followed by MetaWRAP bin refinement. Within MetaWRAP, CheckM [40] was used to determine completeness and contamination of the resulting genomes, retaining only those passing the quality score (QS, calculated as completeness - 5 * contamination) of 50 or above (QS50) [41]. GUNC [42] was used to identify potentially chimeric bins, excluding bins with contamination >0.05, clade separation score (CSS) >0.45 and reference representation score (RRS) >0.5 - in other words, bins containing contigs potentially belonging to more than one taxonomic group. The final set of MAGs (n=188) were clustered using dRep [43], at an average nucleotide identity (ANI) of 95% and alignment fraction (AF) of 30%. The species representative for each genome cluster was taxonomically annotated using the Genome Taxonomy DataBase release 202 (GTDB) [44] and toolkit (GTDB-tk) [45]. This resulted in a final MAG catalogue of 11 species representative genomes from the three HoloFood trials. Using the marker gene multiple sequence alignment from GTDB-tk, a maximum likelihood phylogenetic tree was generated with IQ-tree [46] using the LG+F+I+G4 model and visualised with iTOL [47]. EukCC2 [48] was used to search for potential eukaryotic genomes in our dataset using the bins obtained from CONCOCT and metabat2 (constituent binners within metaWRAP) as input. However, no eukaryotic genomes were identified. In addition, PathoFact [49] was run on the *Pseudomonas aeruginosa* genome to facilitate the identification of potential virulence factors, specifically the toxin and virulence subworkflows.

### Estimating species abundance using the MAG catalogue

Sample reads which passed quality control were mapped to the salmon MAG catalogue with bwa-mem and samtools [35, 36] to assess the fraction of reads that could be accounted for by the catalogue. Reads were considered mapped to a genome if they uniquely matched a genome with at least 90% ANI and $\ge$60% of the read was aligned to the genome. A genome was considered present in a sample if 30% of the genome was covered by the mapped reads. Relative abundance was computed from the total mapping counts (here we omitted the 30% genome coverage requirement to negate the large number of 0 s in the data) and calculated as the number of sample reads mapped to a genome, normalised by both the paired read count (divided by 1 M) and by the genome length (divided by 1 M). This is an adaptation of the RPKM formula [50], hereby referred to as RPMM (Reads Per Million bases of genome, per Million mapped reads). A log_10_ normalisation of RPMM was taken to enable visualisation of relative abundance across the catalogue. A PCA was generated to assess the statistical differences between MAG composition in samples. Read counts mapped to MAGs were normalised using the centred-log ratio using the microbiomeR [51] package and plotted with phyloseq’s plot_ordination function [52]. A PERMANOVA was performed using the adonis function from the vegan package [53] with the bray-curtis distance measure for metadata types: trial and time point. A pairwise ANOSIM within each metadata type was also performed to identify the groups causing significant differences in microbial composition.

### Comparison with other catalogues

The HoloFood MAG catalogue was compared to two existing fish gut genome catalogues: MGnify non-model fish gut genome catalogue - version 1 (hereafter referred to as “MGnify catalogue”), and the Salmon Microbial Genome Atlas (SMGA) [54] which is derived from a mixture of wild and farmed freshwater and saltwater salmon samples. The SMGA samples are primarily long-read sequenced isolate genomes (n=131), complemented with MAGs (n=80). The same QS50 threshold (described above) was applied to both catalogues for the purposes of comparison. To understand the novelty of these two catalogues compared to the HoloFood MAG catalogue, all the HoloFood MAGs were de-replicated with the MGnify and SMGA catalogues. Species representative genomes for clusters comprising at least one genome derived from a salmon gut sample (as MGnify catalogue also contains non-salmon samples) were taxonomically annotated with GTDB-tk and a maximum likelihood tree was generated with IQ-tree and plotted with iTOL.

### Stratification of *Mycoplasmoidaceae* species genomes

Multiple approaches have been used to analyse the genomic diversity within species, from simple ANI based approaches to single nucleotide variant population analysis, but neither approach fully exploits the information coming from core and accessory genomic variation. In contrast, POPulation Partitioning Using Nucleotide Kmers (PopPUNK) employs an alignment-free kmer based approach to define core and accessory genome sequences. The core-accessory distances are used for interrogating sub-species diversity. PopPUNK was used to generate sub-species clusters using the lineage model - a nearest neighbour method, which creates a network of genome clusters based on the closest core distances. The rationale of using this method to predict clusters is due to the small number of genomes in the dataset. The pairwise k-mer distance distributions are dispersed, and fitting a model to this distribution cannot confidently place the genomes in clear networks. Consequently, the lineage method does not attempt to fit a model by k-mer distance, but adds the nearest neighbours to a network - defining connected components as potential lineages [55].

### Multi-kingdom catalogue generation

VIRify [56] was used to predict viral contigs in all assemblies and co-assemblies produced by the HoloFood data. The resulting annotated contigs were analysed for the presence of mobile genetic elements (MGEs) using a mobilome annotation pipeline (MAP) https://github.com/EBI-Metagenomics/mobilome-annotation-pipeline.git. MAP runs geNomad to further identify viral and prophage sequences. To retain good quality predictions, MAP refines the viral predictions from VIRify using CheckV [57] to exclude those predictions lacking viral genes, quality defined as ‘Not determined’ or k-mer frequency >1.0. All high quality CheckV predictions were retained. Additional geNomad viral predictions are filtered to keep annotations with a viral score $\ge$0.8. The filtered viral sequences were clustered using an all-against-all BLAST followed by UCLUST to cluster sequences at 95% ANI and 85% coverage as recommended by CheckV and the Minimum Information about an Uncultivated Virus Genome (MIUViG) standards for viral genomes [57, 58]. Plasmid sequences were clustered using the same method but the thresholds were modified to account for fragments, instead of incomplete plasmid sequences. We used 80% ANI to identify highly similar plasmids, a value used for replicon typing [59] and 85% coverage suggested for incomplete plasmid fragments [60]. The non-redundant set of plasmid sequences were mapped to the PLSDB plasmid database using BLASTn with the same identity and coverage thresholds used for clustering [61, 62].

## Results and discussion

### Genome-resolved metagenomics reveals low prokaryotic species diversity in the salmon gut, dominated by sub-species belonging to the *Mycoplasmoidaceae* family.

#### HoloFood MAG catalogue

A prokaryotic genome catalogue was generated using shotgun metagenomic sequencing of 538 samples taken from the distal gut of farmed Atlantic salmon. All of the gut content samples were mapped to the salmon host genome, and contained on average 95.5% host DNA for trial A samples, 94.9% for trial B samples and 99.0% for trial C samples, resulting in a low number of microbial reads for assembly. After removal of host and low quality sequences, read counts ranged from 698 paired reads and 17.5 million paired reads, with an average of 1.6 million paired reads per sample. Assembly and co-assembly were performed as described in the methods section, resulting in 515 assemblies producing contigs of sufficient quality for binning. The single-run assemblies were individually binned and generated a combined total of 166 genomes that passed the QS50 threshold. The co-assemblies contributed as follows: (i) co-assembly of runs (*n=276*) that produced contigs yet no bins after MetaWRAP bin-refinement contributed 4 additional bins; (ii) co-assembly of trial A samples contributed 4 additional bins; (iii) co-assembly of trial B samples contributed 7 additional bins; (iv) co-assembly of all trial A and trial B samples contributed 7 additional bins. Combined, the single and co-assemblies yielded 188 bins that passed the QS50 threshold and were not filtered out by GUNC, and all bins had less than 5% contamination.

**Fig. 1:**
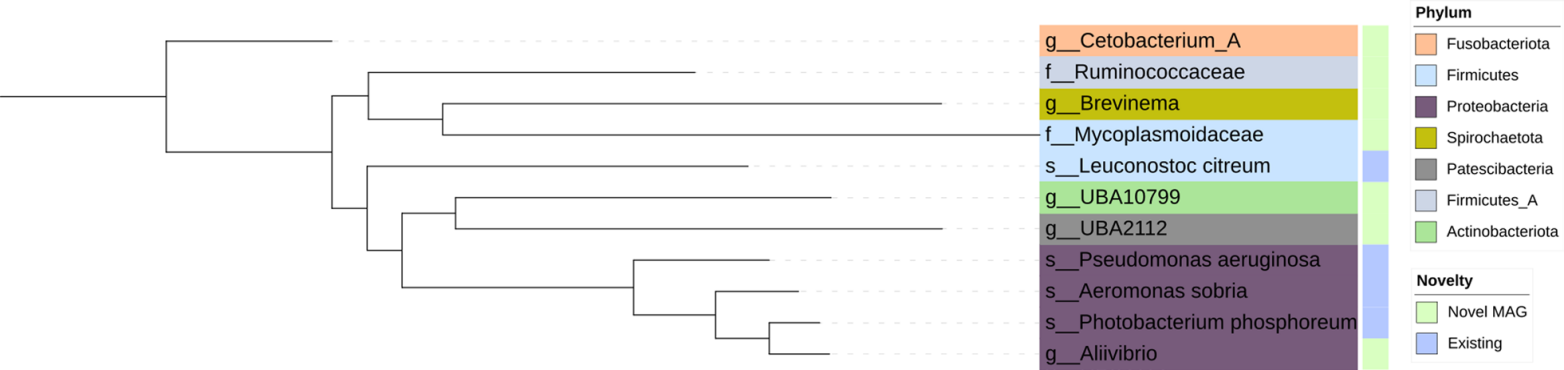
The HoloFood MAG catalogue - Maximum-likelihood phylogeny of the 11 species representative MAGs. Branch tip labels are indicated by the last taxonomic level annotated by GTDB-tk, with the corresponding phyla indicated by colours. The bar on the right hand side indicates novelty with regards to GTDB. Novel species are green and existing species in blue

De-replication of the 188 bins at the species-level produced 11 species representative MAGs (Fig. 1), of which 3 representative genomes were derived from co-assemblies. The 3 genomes (*Leuconostoc citreum *and a novel species from each of the genera *UBA10799* and *UBA2112*) are species representatives of clusters containing 2-3 genomes that were only generated from co-assembly bins. Co-assembly derived bins are present together with single-assembly derived bins in 5 species clusters including two of the largest clusters, a species belonging to the family *Mycoplasmoidaceae *and a species belonging to the genus *Brevinema* (Additional file 3, Fig.1). Hereafter the word novel is used to describe a genome without a species annotation with respect to GTDB. In total, 4 genomes match to existing species and 7 are novel species, annotated to the genus or family levels. Hereafter novel species will be referred to in the text with the suffix “.*nov”*. As defined by CheckM [40], 6 genomes are near-complete and the remainder have >50% completeness and $\le$5% contamination. By MIMAGs standards [63], one genome is a high-quality draft genome - defined by the presence of sequences encoding the ribosomal RNAs (rRNAs) and tRNAs for at least 18 amino acids, and 10 genomes are defined as a medium-quality, i.e. passing completeness and contamination scores but without the presence of the required marker genes. Resolving full length rRNAs is a known challenge in short-read metagenomic data. In the *Mycoplasmoidaceae.nov* cluster, the co-assembled genomes have a QS between 68-72, which is lower than the single-assembled genomes with quality scores mostly between 80-94. This lower quality can be accounted for by the intraspecies variation resulting in fragmented co-assemblies. Conversely in the *Brevinema.nov* cluster, one co-assembly has a low score of 50.86 and the other 3 co-assemblies score as 88.76, 92.13 and 92.13, similar to the single-assembly genomes in this cluster, with the representative being derived from single-run assemblies (Additional file 3, Fig. 1).

#### Sample diversity and MAG abundances

During the post quality control we discarded two samples lacking sufficient reads, while reads from the remaining 538 samples were each mapped to the HoloFood MAG catalogue to determine the proportion of the 11 species representative genomes found in each sample (Additional file 3, Fig. 2). The runs with very low read-counts have variable mapping percentages between 10-85%. As the read-depth of the samples increases, there is an overall increase in the percentage of reads mapped, however this still varies between 50-90%. After 7.5 million reads the mapping percentage remains above 75%. To understand the effect of read depth on genome resolution, we applied a stricter mapping threshold. A species was considered present only if reads from a sample covered at least 30% of the genome to which they were mapped. (Additional file 3, Fig. 2). The majority of samples map to one species, the *Mycoplasmoidaceae.nov *genome. No correlation was found between the read count per sample and the presence of more than one genome species in the sample. This result indicates that the microbial community is dominated by one or two species and increased read depth would likely not result in further resolution of new species level genomes.

**Fig. 2:**
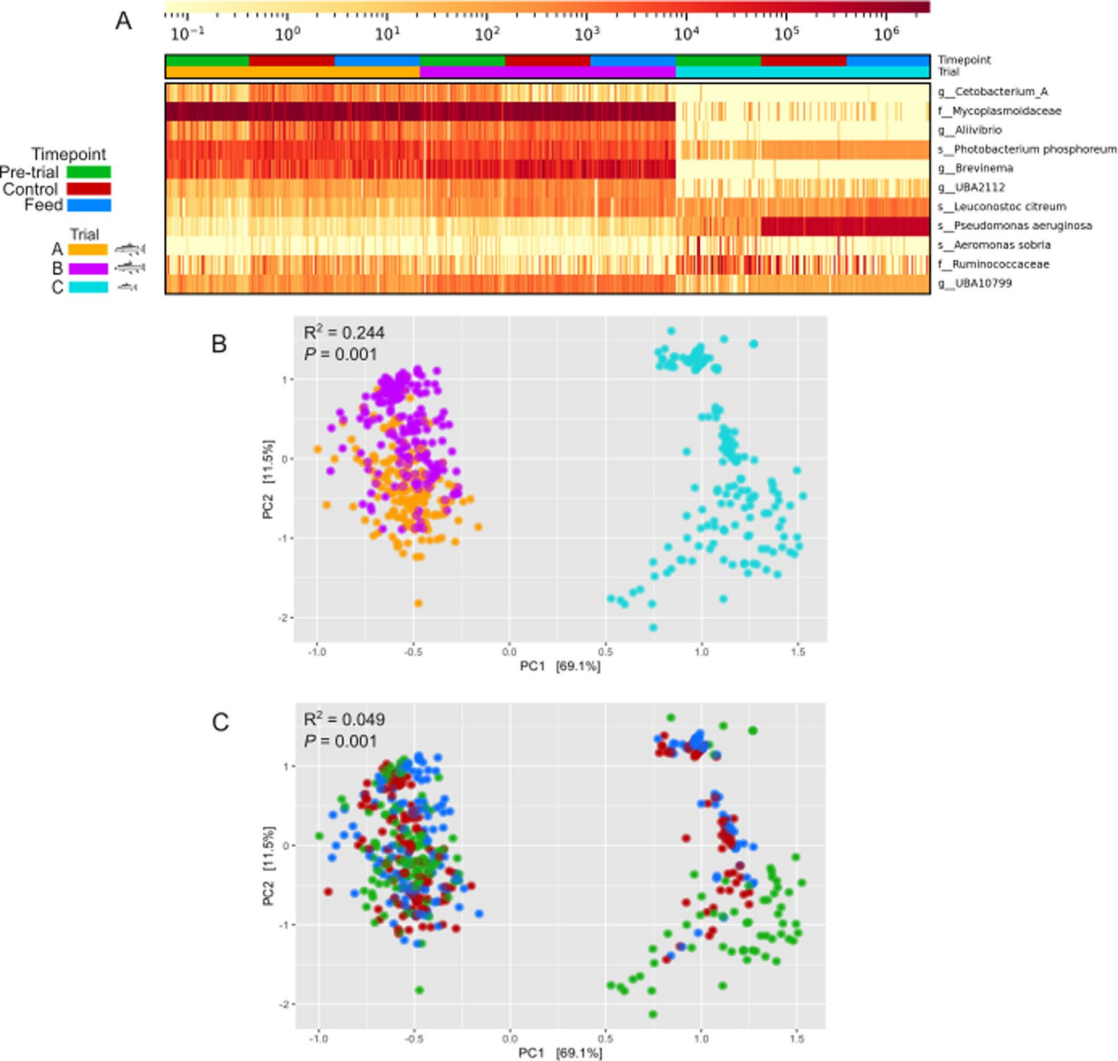
Relative abundance of each sample mapped to the HoloFood MAG catalogue. Samples are grouped by trial (A, B or C). Pre-trial samples are taken on day 0 of the trial. Control and feed refer, respectively, to the control and test diets for samples taken at the end of each trial. B - PCA of the counts of samples mapped to MAGs coloured by trial with PERMANOVA results. C - PCA of the counts of samples mapped to MAGs coloured by time point with PERMANOVA results

To complement the read-mapping, we computed the relative abundance of each MAG, referred to as RPMM, which is the mapped read count per sample normalised by the total read counts and genome length (Fig. 2C). A PCA of read counts per sample mapping to the MAGs shows similar separation of microbial composition for trials A and B, and a separate grouping for trial C. According to a PERMANOVA, the trial explains 24.4% of the compositional variance. Pairwise ANOSIM for each trial type (A-C: R2=0.870, P=0.001, B-C: R2=0.834, P=0.001, A-B: R2=0.025, P=0.001) shows that samples in trial A and trial B have very little compositional difference. Trial C is responsible for the compositional difference between trials, and as this trial contains salmon of a different development stage we cannot conclude that microbial composition is defined by the feed (Fig. 2B). *Mycoplasmoidaceae.nov* is the most abundant species across samples in trials A (RPMM: M=1.83M, SD=0.76M) and B (RPMM: M=1.33M, SD=0.71M). It is less abundant in trial C (RPMM: M and SD are near 0). The next most abundant species is *Photobacterium phosphoreum* which are dominant in trial C (trial A and trial B RPMM: M and SD are near 0, and trial C RPMM: M=0.12M, SD=1.00M). In trial C the overall microbiome composition is significantly different to trials A and B (Fig. 2A), whereby *Pseudomonas aeruginosa *(trial A and trial B RPMM: M and SD are near 0, and trial C RPMM: M=0.12M, SD=0.10M) is the most abundant in fish at the final time-point (day 77) in both the control and test diets. The pre-trial samples for trial C, taken at day 0, have a visibly lower abundance of *Pseudomonas* *aeruginosa *(Fig. 2A). Fig. 2C confirms that most pre-trial samples taken on day 0 have a slightly different composition to control and feed samples taken at day 77, however there is still overlap at all timepoints for some samples. Unlike trials A and B, *Mycoplasmoidaceae.nov *in trial C has a lower abundance and is even undetectable in 139 of 180 samples. *Pseudomonas* *aeruginosa *and *Aeromonas* *sobria *(trial A and trial B RPMM: M and SD are near 0, and trial C RPMM: M=0.01M, SD=0.04M) obtained from trial C, were too low in abundance in the other trials to assemble into genomes, but were still detected in samples. *Pseudomonas* *aeruginosa *was detected in 492 samples and *Aeromonas* *sobria* in 148 samples across all trials.

Low microbial diversity is commonly encountered in fish microbiome studies. The HoloFood data additionally have a high percentage of host contamination due to the sampling of the whole distal gut, further reducing the microbial reads available to recover genomes. To address this, the reads were co-assembled with the aim of recovering additional low-abundance species. Co-assembly successfully added 3 species to the catalogue, and these genomes were found in between 445 to 518 samples across all trials but at a near-0 RPMM in comparison to the dominant species above, explaining why they were not recovered from single-run assemblies. The low diversity of the fish gut combined with a few dominant species has also been observed in prior 16SrRNA metabarcoding and shotgun metagenomic studies [9, 10], particularly in salmonids and Atlantic cod [11]. In agreement with our study, the dominant species has typically come from the genus *Mycoplasma *which is considered to be a symbiont adapted to live inside the gut epithelial cells of the salmon host with a reduced genome size and gene content as a result of its co-evolution with the host species [15, 18, 64]. However, this same *Mycoplasmoidaceae.nov* species has a lower abundance in trial C samples, indicating that the age of the fish and/or the environment impacts the microbiome, with a more stochastic microbiome composition in juvenile fish having less developed immune regulation. Previously, Dehler et al. [64] identified that *Mycoplasma* were more dominant in fish residing in seawater because *Mycoplasma* relies on host-produced cholesterol and other sterols for nutrition, which is more available when the fish host has increased levels of lipolysis during smoltification - the migration of juvenile fish from freshwater to seawater [65]. A less diverse microbiome has been observed in migratory juvenile salmon, with only returning adults having a stable microbiome dominated by *Mycoplasmataceae * [8]. While the presence of the dominant symbiont may be considered detrimental, the absence of this microbe is often associated with a take over of opportunistic pathogens such as the *Pseudomonas aeruginosa *that then dominate the fish microbiome [4, 66, 67]. It is important to note that *Pseudomonas aeruginosa* is a common microbe, particularly in marine and built environments [68, 69]. This species is present at high abundance in different HoloFood samples taken from different tanks. This prevalence and abundance is suggestive that it is not ad hoc contamination, and is part of the salmon gut microbiome. In later sections, we used PathoFact to identify potential evidence of pathogenicity.

#### Comparison with other salmon gut catalogues

**Fig. 3:**
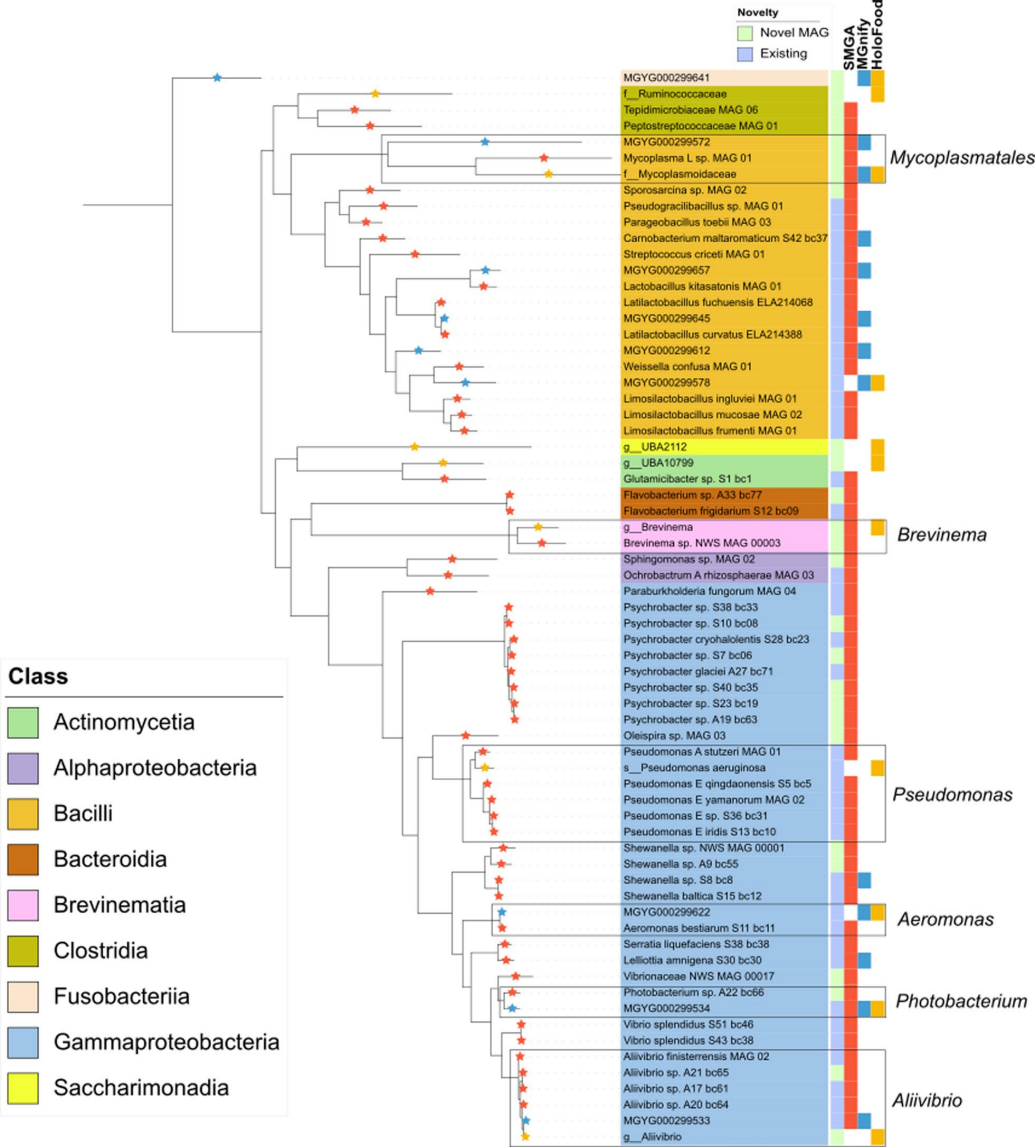
Maximum likelihood phylogenetic tree of representative bacterial species from a range of salmon samples from three sources. Coloured labels on the right show in order: genome novelty and presence of cluster members in the three catalogues SMGA, MGnify and HoloFood. Membership of the cluster representatives are indicated as stars on the branches (left) and follow the same colour scheme

The genomes used to generate the HoloFood salmon catalogue (*n=188)* were compared against two other fish datasets: the MGnify non-model fish gut catalogue v1.0 (filtered for species clusters comprising at least one salmon sample *n=9*), and SMGA with QS $\ge$50 (*n=198)* (Fig. 3). The HoloFood and SMGA catalogues share only 3 genomes: *Mycoplasmoidaceae.nov, Brevinema.nov *and *Photobacterium phosphoreum*, while the HoloFood and MGnify catalogues share five genomes: MGYG000299641 (*Cetobacterium_A*), *Mycoplasmoidaceae.nov, *MGYG000299578 (*Leuconostoc citreum*), MGYG000299622 (*Aeromonas sobria)* and MGYG000299534 (*Photobacterium phosphoreum)*. Except *Photobacterium phosphoreum*, the species clusters shared with the MGnify catalogue represent genomes that are also found in non-salmon fish species. Notably, the abundant novel *Mycoplasmoidaceae* genome found in the HoloFood catalogue is common in all three catalogues, suggesting that the species reconstructed from the HoloFood dataset is a wide spread symbiont in the salmon and other fish microbiomes. There are five genome clusters unique to the HoloFood dataset, of which four are novel species. Since this work, the MGnify non-model fish catalogue has been updated to incorporate the novel diversity found in the HoloFood dataset.

The nine MGnify salmon microbial genomes were shared with one or both of the other catalogues, whereas the SMGA catalogue contains many unique genomes, particularly in the class Gammaproteobacteria. One large Gammaproteobacteria clade specific to the SMGA catalogue are *Psychrobacter* species found in both fish and environmental samples. The three genomes that were annotated to a species level, were previously identified in marine or coastal ice samples: *Psychrobacter glaciei* was isolated from an ice core of an Arctic glacier [70], and *Psychrobacter* *cryohalolentis *isolated from highly saline region of permafrost [71]. Similarly, the clade in between *Pseudomonas* and *Aeromonas* belongs to the genus *Shewanella*. One genome is annotated as *Shewanella baltica, *found on freshly caught fish and is able to survive the low temperatures of laboratory storage conditions [72]. The remaining SMGA Gammaproteobacteria genomes mostly belong to the same genus or a closely related genus of the nearest MGnify and HoloFood genomes. For example, the *Pseudomonas *clade contains *Pseudomonas* species from the genus *Pseudomonas_E*, of which three can be traced to environmental samples: *Pseudomonas_E yamanorum* isolated from a subantarctic soil sample [73], *Pseudomonas_E qingdaonensis *isolated from soil rizosphere [74] and one *Pseudomonas_E iridis* found in a 16 S study of fish farms in Turkey [75]. The closest related genomes for the HoloFood *Pseudomonas aeruginosa* are from marine and fish environments which further confirms that this is unlikely to be a contaminant species from sampling procedures. The *Aeromonas* clade contains two species of *Aeromonas sobria *and one *Aeromonas bestiarum*, a common fish pathogen. The *Photobacterium* and *Aliivibrio *clades indicated are all species of the same genus. Two classes, Bacteroidia and Alphaproteobacteria, contain SMGA species only. Bacteroidia comprises two *Flavobacterium* species, associated with disease in fish [76], and *Alphaproteobacteria* comprises a *Sphingomonas *species and *Ochrobactrum_A rhizosphaerae* found in the potato rhizosphere environment.

We mapped the HoloFood reads for each sample to the SMGA catalogue as described previously. The number of mapped reads does not significantly increase for any of the samples in comparison to mapping to the HoloFood MAG catalogue, suggesting that the SMGA collection does not contain additional genomes that can be found in the HoloFood samples. The increased number of genomes (and hence diversity) in the SMGA catalogue compared to HoloFood can be attributed to the environments which the fish were sampled from and that many of the genomes were derived from whole genome sequencing of pure isolated cultures. The SMGA catalogue comprises both farmed and wild fish in freshwater and seawater. Wild fish are migratory, and experience radical environmental changes between the juvenile and adult life stages, where environmentally sourced microorganisms likely explain the significant compositional differences between juvenile and adult salmon [8, 21]. In contrast, the fish farmed in the HoloFood project come from highly controlled and similar environments. Nevertheless, our new HoloFood catalogue presents a detailed representation of numerous distinct populations of the salmon associated *Mycoplasmoidaceae* species, also present in the other catalogues, further highlighting the ubiquitous presence of this taxon in the fish gut.

### Pan-genome analysis of genomes within the HoloFood catalogue

The HoloFood MAG catalogue is made up of 11 species representatives, of which only one genome – *Aeromonas sobria *is a singleton cluster, demonstrating our ability to reliably recover similar, but distinct genomes. Five species clusters are composed of more than 10 genomes comprising at least one genome with $\ge$95% completeness. The *Mycoplasmoidaceae* species is the largest cluster made up of 79 genomes, followed by *Brevinema.nov* (48 genomes), *Pseudomonas aeruginosa *(24 genomes) and *Photobacterium phosphoreum *(13 genomes). *Mycoplasmoidaceae.nov *species are common and highly abundant in the Atlantic salmon gut microbiome, and all existing metagenomic studies have resolved at least one species per study. As *Mycoplasmoidaceae.nov* is also the largest species cluster in the HoloFood dataset and is shared with other public catalogues, we used the core and accessory genes to separate this species cluster into sub-species. The pan-genome tool Panaroo [77] was used to compare the core and accessory genes between genomes. To avoid introducing biases of incomplete genomes and small species clusters, we investigated the pan-genomes of 3 clusters including the *Mycoplasmoidaceae.nov* cluster, comprising at least 5 near-complete genomes ($\ge$95% completeness). The *Mycoplasmoidaceae.nov* species cluster has 36 near-complete genomes for which Panaroo identified 1,036 genes present in $\ge$95% of genomes (core) and 404 genes present in <95% of genomes (accessory). The number of accessory genes are less than half of the core genes indicating that the genomes are closely related. The aforementioned approach was also applied to *Pseudomonas aeruginosa *and *Photobacterium phosphoreum. *The number of genes in *Mycoplasmoidaceae.nov *are also lower than other genomes in Table. 1, which reflects the small genome size of *Mycoplasmoidaceae.nov*.

**Table 1:** .

Species representative	Genomes	Genomes $\ge$95% complete	Core genes	Accessory genes	Total genes
*Mycoplasmoidaceae.nov*	79	36	1,035	404	1,439
*Pseudomonas aeruginosa*	24	11	5,413	913	6,326
*Photobacterium phosphoreum*	13	6	4,100	155	4,255

Core and accessory genes as predicted by Panaroo for clusters with 5 or more near-complete genomes, filtered by $\ge$95% completeness. Core is defined as present in $\ge$95% of genomes, and accessory as present in <95% of genomes

### Exploration of *Mycoplasmoidaceae* at a sub-species resolution

**Fig. 4:**
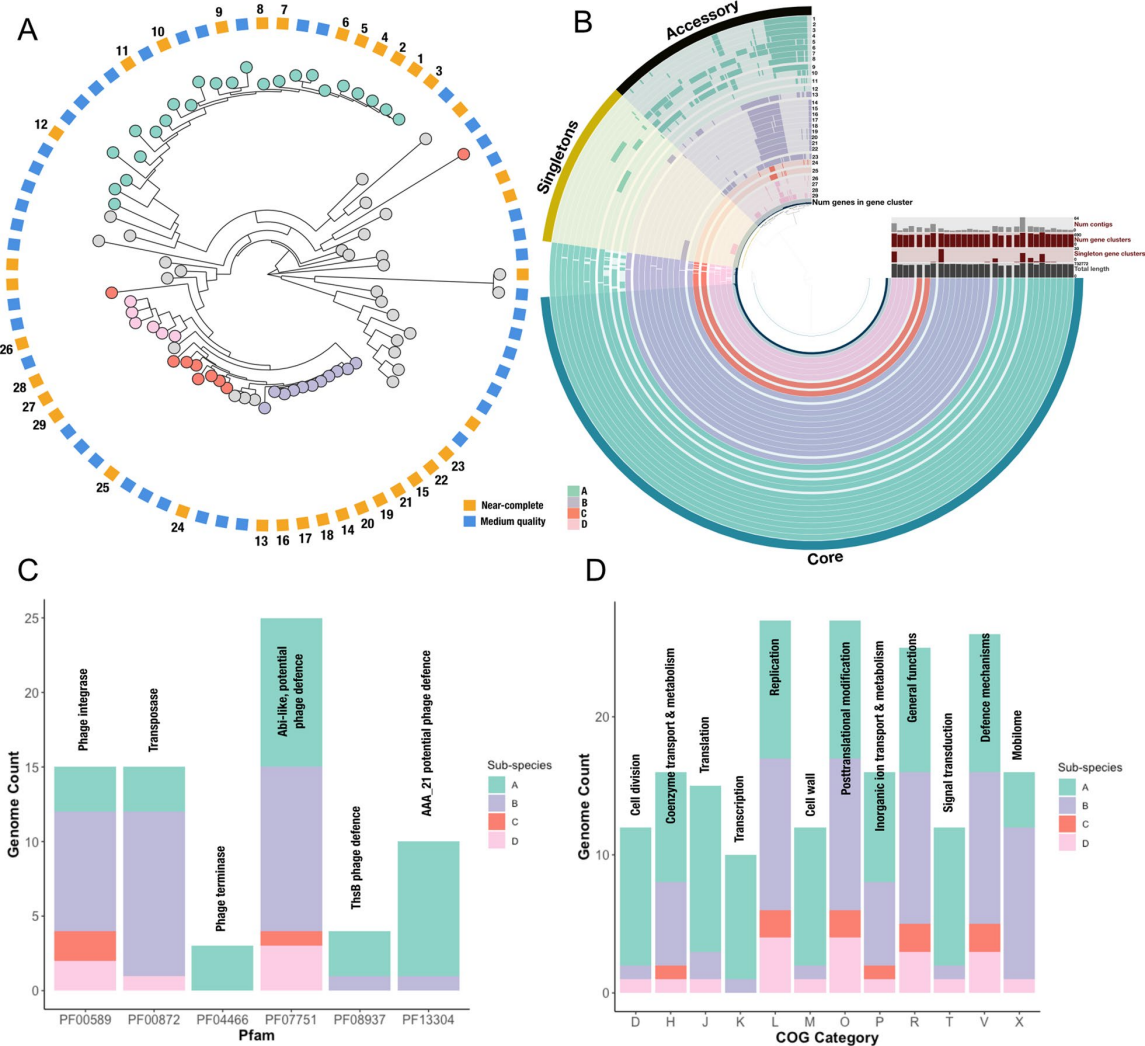
Separation of *Mycoplasmoidaceae.nov* genomes. A - PopPUNK tree of 79 genomes. The outer ring indicates genome completeness. The colour of the nodes indicate the sub-species estimated by PopPUNK. Nodes for which the PopPUNK placement was deemed inconclusive are coloured grey. B - Pan-genome for a subset of 29 of the near-complete genomes clustered by similarity of gene clusters. The genomes are coloured by PopPUNK sub-species clusters and numbered according to their placement in the PopPUNK tree. C - Number of genomes with accessory gene clusters annotated with a Pfam relating to phage or mobilome. D - Number of genomes annotated with accessory gene clusters annotated with a COG category

To explore the population structure of the *Mycoplasmoidaceae.nov* species further, we first de-replicated the species cluster using dRep at 99% ANI and 60% alignment fraction to obtain potential strain level separation. However, this approach did not subdivide the species cluster, and separation was only observed at minimum 99.7% ANI. A uniform ANI for sub-species separation is not well-defined, but *Mycoplasmoidaceae.nov* is made up of small, very similar genomes. We used PopPUNK [55] to identify sub-species based upon shared core and accessory nucleotide sequences. The same subset of 36 *Mycoplasmoidaceae.nov* genomes with >95% completeness were used as input to PopPUNK, which was run using the ‘lineage’ method to separate the genomes into a network of four potential clusters (Fig. 4A). This clustering was subsequently used to place the remaining 43 medium quality MAGs in the network.

The PopPUNK-assigned cluster was retained where it was supported by at least two near-complete genomes, and most near-complete genomes belonged to a monophyletic group. The remainder were deemed inconclusive (coloured grey in Fig. 4A). Using this approach, four sub-species of *Mycoplasmoidaceae.nov *are estimated in the HoloFood dataset. The pan-genome of near-complete genomes belonging to each of these 4 sub-species was calculated using Anvi’o [78, 79] (Fig. 4B). The pan-genome has 815 gene clusters in total, of which 531 gene clusters are shared across all *Mycoplasmoidaceae.nov *- labelled as core genes, and 109 gene clusters belong to only one genome and are labelled as singletons. The remaining 139 gene clusters are labelled as accessory and are present across the genomes in different combinations and separate the sub-species (Fig. 4B). PopPUNK confidently separates sub-species A from the others and this is supported by the large number of accessory genes uniquely present in its pan-genome, including one clear segment of prevalent gene clusters in genomes 1-9. PopPUNK defines sub-species B to comprise a monophyletic clade (genomes 14-22) and two out-groups (genomes 13 and 23) in Fig. 4A. This is once again supported by the pan-genome, as genomes 14-22 share two similar segments of accessory genes and have a consistent number of genes across a similar genome length. Genomes 13 and 23 also share the same gene clusters but have additional unique accessory and singleton gene clusters. Finally, PopPUNK estimates sub-species C and D to be phylogenetically similar to sub-species B. These sub-species have fewer genes per genome, and most of the accessory genes are shared with sub-species B confirming the placement of PopPUNK. The contig numbers in the *Mycoplasmoidaceae.nov* genomes range from 4 to 64 contigs, and more fragmented assemblies affect gene prediction and consequently pan-genome analysis. However, as PopPUNK sub-species are derived from the genome sequence alone, the predicted sub-species are independent of gene prediction.

Given the close relationship between the sub-species and the pattern of accessory genes, the Anvi’o accessory gene clusters were functionally annotated with clusters of orthologous genes (COG) categories and Pfam. Many gene clusters could not be functionally annotated - 46 gene clusters annotated with Pfam and 32 annotated with COG categories. All 4 sub-species encode Pfam domains or families related to phage and transposon activity (Fig. 4C). Sub-species C only has presence of phage integrase and phage defence, sub-species B additionally has mobile elements - transposons from the mutator family and sub-species A and B also had presence of phage terminase and two additional phage defence mechanisms. All additional functions were summarised by their COG categories (Fig. 4D). Similar to the Pfam results, all sub-species comprise defence mechanisms and sub-species A, B and D have elements of the mobilome. Notably, sub-species C, which has a reduced number of accessory gene clusters, does not encode for some vital housekeeping functions such as cell division, transcription, translation, cell membrane biogenesis and signal transduction, but at least one of the genomes has retained functions relating to metabolism. Loss of functional genes was previously observed in intracellular *Mycoplasma* in Atlantic salmon hosts [16]. Additionally, the metabolic potential of *Mycoplasma* MAGs resolved from salmon metagenomes has been studied extensively by Rasmussen et al. 2021 [15] and Rasmussen et al. 2023 [17] who concluded a loss of basic functions such as the citric acid cycle. Instead, glycolysis and vitamin and cofactor metabolism e.g. thiamine biosynthesis were commonly identified pathways in the MAGs. Recovered *Mycoplasma *genomes were smaller in size, and appear to be dependent on the host for nutrients and survival functions, and the species were considered obligate parasites.

Using PopPUNK we are able to demonstrate the presence of *Mycoplasmoidaceae.nov* sub-species in the HoloFood dataset. The pan-genome shows the presence of different accessory gene clusters in the sub-species, confirming the initial prediction of PopPUNK. We identify shared phage defence and phage integration functions in all sub-species, which indicates current or previous bacteriophage infection. However, whilst genomic results confirm the presence of genes, transcriptomics would be needed to determine if the genes are actively expressed. We also identify reduced genes and loss of gene function for one sub-species D, which is common in intracellular obligate parasites.

### The virome and mobilome

#### The virome

**Fig. 5:**
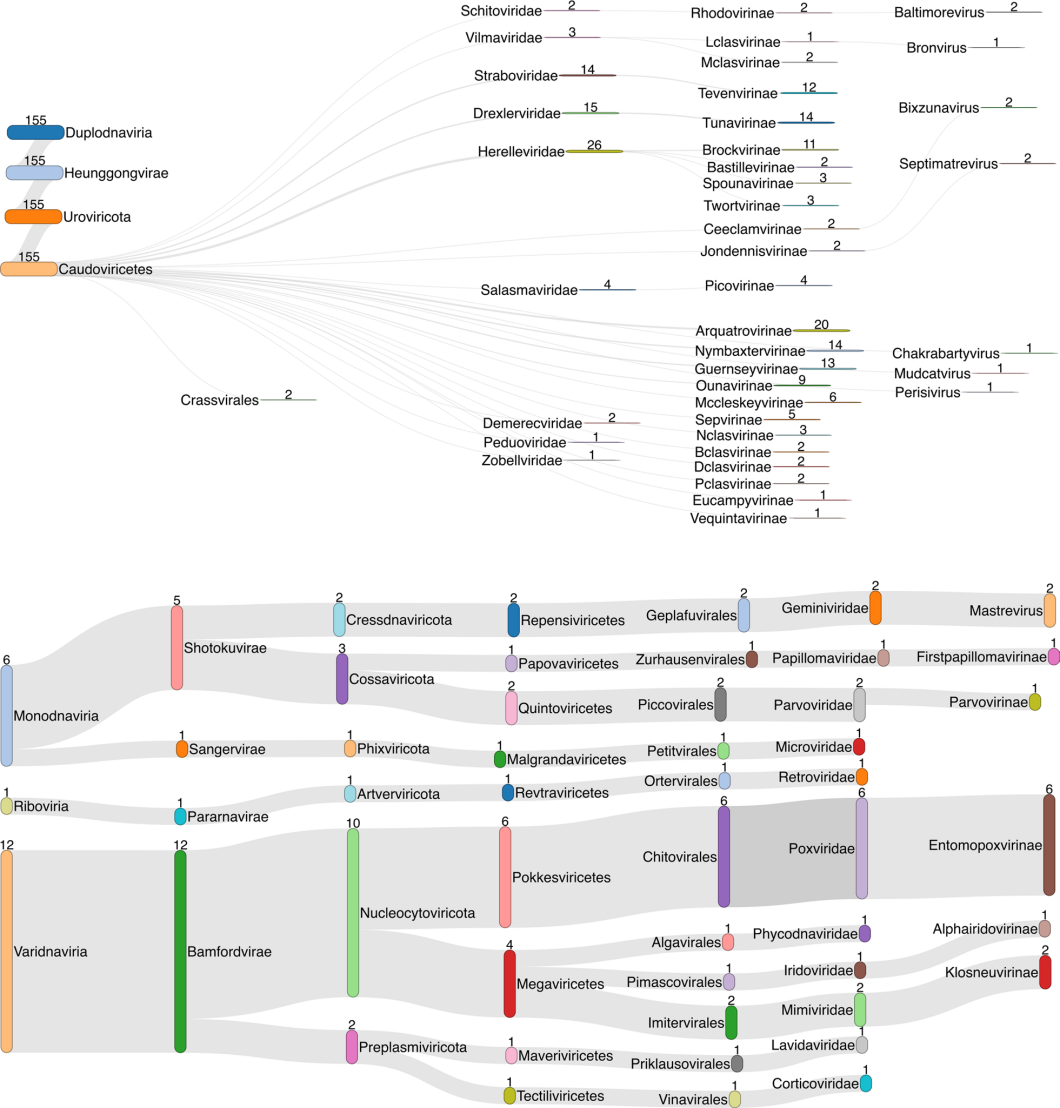
Taxonomic profile of viral sequences predicted in assemblies with 302 Caudoviricetes-unclassified sequences and 1,836 unclassified sequences omitted to aid easier visualisation

VIRify and geNomad predicted 7,394 viral sequences and 280 prophage sequences from 163 salmon single-run assemblies and the four co-assemblies. The remaining single-run assemblies did not yield any viral annotations. After clustering at 90% ANI and 85% coverage, we obtained a non-redundant set of 2,313 viral sequences and 18 prophage sequences. We identified 170 of the 280 unclustered prophage sequences in the HoloFood genomes: *Pseudomonas aeruginosa *(n=115), *Brevinema.nov* (n=56), *Aliivibrio.nov* (n=5), *Ruminococcaceae.nov* (n=2), *Photobacterium phosphoreum *(n=8). We did not find the presence of prophage in the *Mycoplasmoidaceae.nov* genomes. Fourteen of the prophage cluster representative sequences belonged to the Caudoviricetes class, 1 *Imitervirales* and 3 unclassified. The majority of viral sequences are classified only up to the Caudoviricetes class (n=302), and 1,836 viral sequences are unclassified. Caudoviricetes is a very large and diverse class of viruses which dominate the existing viral databases with genomic sequence data, therefore it is not uncommon to have many classifications [80, 81]. The remaining taxonomy is shown in further detail in Fig. 5. *Bicauviridae* are archaeal viruses of which 12 cluster representatives belong to *Bamfordvirae*. Within this kingdom, the phylum *Nucleocytoviricota* [82] is typically found in marine environments with eukaryotic algae hosts. Similarly, in the phylum *Preplasmiviricota *we identify *Lavidaviridae,* commonly known as virophages found in marine algal hosts, co-occurring with *Mimiviridae, *which are giant viruses infecting marine protozoa amoeba [83, 84], and *Corticoviridae* isolated from seawater [85]. In the *Monodnaviria* branch, *Sangervirae* only infect bacterial hosts, with *Microviridae* being a family of bacteriophages and among the smallest of DNA viruses. *Shotuokuvirae *on the other hand is noted to cause disease in canine, feline and porcine animal hosts.

#### Plasmids and resistome

We identified 2,097 plasmid sequences in the single-run assemblies across the three trials: Trial A (n=303), Trial B (n=176) and the majority were found in the gut microbiomes of juvenile fish from Trial C (n=1,053). Five hundred and sixty five plasmid sequences were identified in the co-assemblies. After clustering, 525 representative plasmid sequences are identified, of which 22 can be matched to a complete plasmid genome in PLSDB (Additional file 1). The majority of matches were plasmids found in *Pseudomonas* or *Vibrio* cells and both genera contain species known as pathogens in aquaculture. However these species can also exist as commensals and are present in a variety of environments and life styles, therefore an assessment of potential pathogenicity is needed. The number of matches was low as PLSDB is made up of complete bacterial plasmid genomes from NCBI, hence finding matches for fragments generated by short-read metagenomic data can be difficult. In addition, metagenomic data will comprise novelty not found in existing isolates. Three hundred and twenty seven plasmids were in HoloFood genomes. After removing redundancy, this was reduced to 67 representative plasmid sequences, demonstrating that we can recover highly similar plasmids or copies. Plasmid sequences present in the genome can have a similar coverage or GC content with the bacterial genome, and this results in the binning algorithm considering them as the same genome. Additionally since only one of the 67 sequences could be matched to an entry in PLSDB we considered the sequences co-binned with the genomes. To assess the effect of co-binned plasmid sequences on the completeness of the genomes, we removed the 327 sequences and recalculated completeness (Additional file 2). This decreased completeness by at most 3.25% and those where completeness decreased more than 2% were still near-complete (>95%). Contamination does not decrease by more than 0.381%, therefore we have retained the sequences in the genomes.

**Fig. 6:**
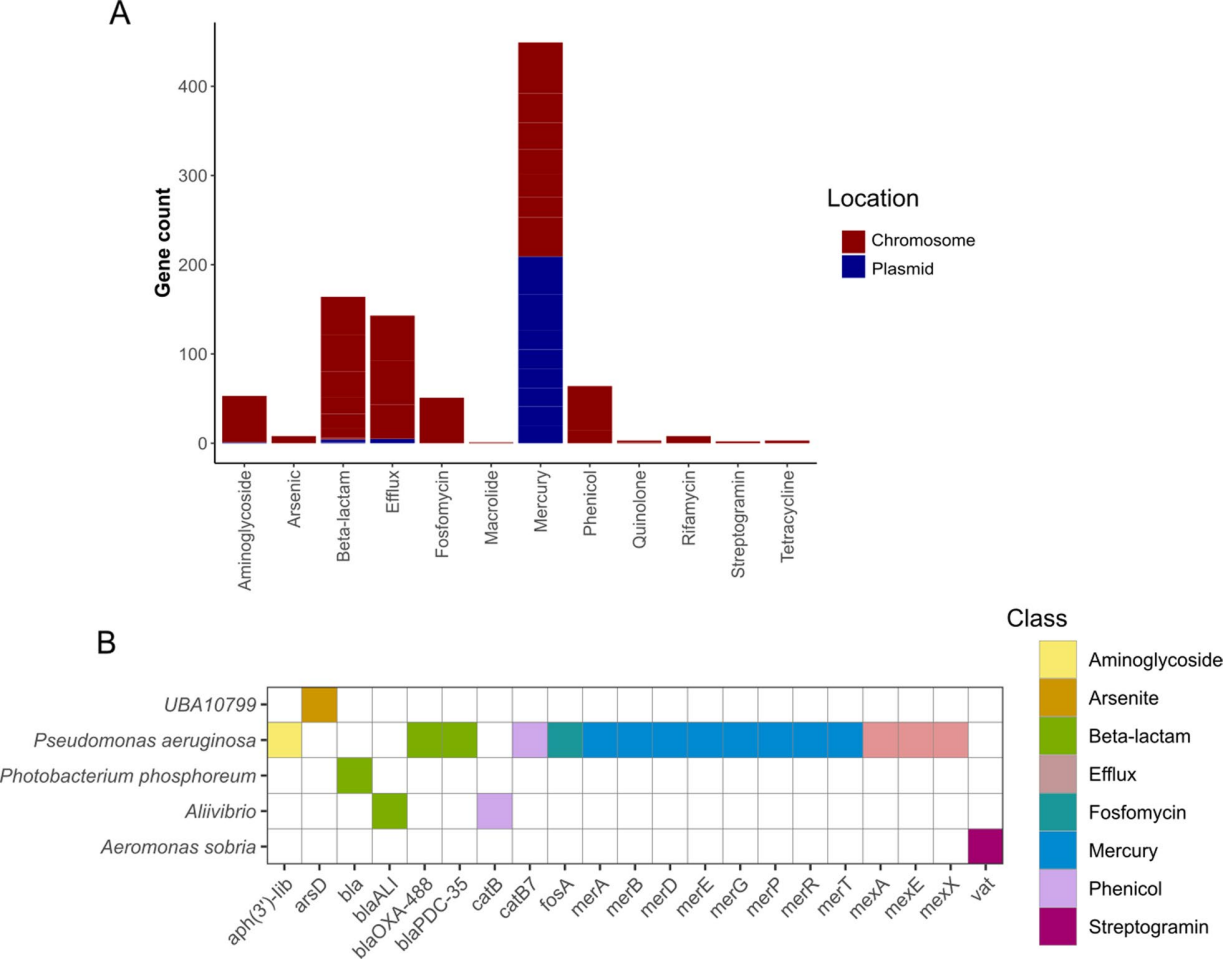
Antimicrobial resistance genes in the HoloFood data across all trials. A - Count of antimicrobial resistance genes in the assembly data and their location. B - Presence/absence of antimicrobial resistance genes in the genomes

We identified a total of 949 antibiotic resistance genes (ARGs), of which 833 were found in the single-run assemblies and 49 in co-assemblies. They are again most prevalent in Trial C: Trial A (n=26), Trial B (n=12), Trial C (n=862). Of the 949 ARGs, 219 were found on a plasmid and 730 labelled as chromosomal, of which 222 are on a contig belonging to a genome (Fig. 6A). *Pseudomonas aeruginosa *carries the largest number of ARGs, and most genes uniquely confer mercury resistance. Heavy metal pollution is introduced into environments through human activities producing industrial waste, and are found contaminating water and soil. An abundance of heavy metals can be toxic to bacterial species, inducing an oxidative stress state and interfering with protein synthesis [86]. As a result, bacteria such as *Pseudomonas aeruginosa *have developed resistance mechanisms to remove toxic mercury from the cell [87, 88]. The most commonly known resistance mechanisms originate from the *mer* operon, identified in the HoloFood data, and can be supplemented by efficient efflux pumps. In addition to metals, we identify clinical antibiotic resistance genes. Antibiotics are used in aquaculture to minimise bacterial infections which lower the fish yield through the spread of disease. Associated resistance to clinically relevant antibiotics in opportunistic bacteria is well documented [89] and pathogenic species from the genera *Aeromonas *and *Vibrio* contribute 43% of the fish pathogens exhibiting antimicrobial resistance (AMR) genes. The HoloFood assemblies contain AMR genes for a range of antibiotics with beta-lactams, chloramphenicols, aminoglycosides and fosfomycin being dominant (Fig. 6B).

When searching for the contigs predicted as plasmid sequences also present in the MAGs, the most prevalent are 16 plasmid sequences found on contigs belonging to the *Pseudomonas aeruginosa *MAG. The largest plasmid sequence in this genome is 106,543bp in length which contains 9 ARGs encoding mercury resistance, and one non-coding region annotated as a mercury resistance transcriptional regulator. Two contigs predicted as plasmids, including the large sequence mentioned, also carry conjugative integrons. PathoFact, used to classify toxin domains and genes, identifies 58 genes with a final toxin confidence level of 1 - indicating the presence of a toxin domain and secretion system. The *Pseudomonas aeruginosa *MAG is the most abundant genome in trial C samples taken at the final time-point, and correlates with a reduced abundance of *Mycoplasmoidaceae.nov. *We can identify the presence of multiple prophage sequences, plasmids, evidence of antimicrobial resistance and toxin genes in the genome derived solely from metagenomic analysis suggesting that the different microbial compositions in the juvenile trial C samples contain a higher proportion of this competitive and putatively pathogenic microbe.

## Conclusions

The HoloFood salmon gastrointestinal catalogue comprises 11 MAGs, demonstrating a low bacterial diversity consistent with previous metabarcoding and metagenomic studies in fish. In the HoloFood samples, the microbiome differs across salmon life stages. Adult salmon are dominated by a single *Mycoplasmoidaceae* species, a known obligate organism, whereas juvenile fish have a higher abundance of *Pseudomonas aeruginosa*. These results are relevant to consider for the design of microbiome interventions for aquaculture at different life stages. The HoloFood catalogue contains unique species to the existing profiled bacterial diversity of salmon, but also shares the *Mycoplasmoidaceae.nov *genome with other catalogues, further suggesting the ubiquitous presence and importance of this obligate organism in the salmon host. We also show evidence of sub-species variation in *Mycoplasmoidaceae.nov*, some of which exhibit reduced genome size and housekeeping functions characteristic of a mutualistic host-microbe relationship. This analysis can be further extended to the existing catalogues of *Mycoplasma *species for other Atlantic salmon or closely related fish microbiome studies, including those known to be internally localised to the cells of the host salmon.

We present the first viral catalogue from fish metagenomic data with evidence of presence of marine infecting viruses and co-occurring giant viruses, a common phenomenon observed in marine protists. However we did not identify any eukaryotic genomes in the HoloFood dataset. In addition, we can identify prophage sequences in the HoloFood genomes, predominantly in *Pseudomonas aeruginosa *that was abundant in trial C. We are able to profile a range of AMR genes and heavy metal resistance genes in the HoloFood samples. The salmon used during the HoloFood trials were not treated with antibiotics and remained in controlled environments, however horizontal transfer of existing resistance genes can persist. Mercury resistance in *Pseudomonas aeruginosa *is an example of this phenomenon, where continued exposure to heavy metals led to some of the earliest findings of mercury resistance in plasmids dating back to the 1970 s [90]. This property of *Pseudomonas aeruginosa* is used as a method for bioremediation to remove toxic mercury waste [91]. These findings highlight the prevalence of antibiotic resistance in the salmon datasets. Increased presence of AMR genes in opportunistic bacterial pathogens is a concern for aquaculture and as such the European Commission has heavily regulated antibiotic usage. There is a need for alternative therapies to prevent widespread infection, such as the use of bacteriophages infecting pathogenic bacteria [24]. Phage therapy requires a collection of bacteriophage sequences present in bacterial genomes or indication of prior infection of a genome through the presence of the antiviral immune defence system, CRISPR-Cas9. We demonstrate that we can identify phage sequences using metagenomics and localise them to genomes of opportunistic bacteria such as *Pseudomonas aeruginosa*. Whilst we were able to identify genes conferring functions relating to pathogenicity using multiple bioinformatics tools, metagenomics alone cannot conclude that the *Pseudomonas aeruginosa* genome resolved in this dataset is pathogenic, which warrants further experimental validation.

## Additional file

Additional file 1: PLSDB matches to 22 plasmids sequence representatives.

Additional file 2: Genome completeness and contamination stats after removal of plasmids sequence.

Additional file 3: Genome mapping and quality figures.

Additional file 4: Experimental designs.

## Data Availability

The datasets supporting the conclusions of this article are available in the HoloFood data portal https://www.holofooddata.org [93], European Nucleotide Archive and MGnify. The metagenomic data (raw reads, assemblies and bins) and MAGs generated during the current study deposited in ENA under accessions PRJEB41657 for trials A and B and PRJEB55376 for trial C. The MGnify metagenomic analyses (Richardson et al., 2023) study accession is MGYS00006041 and the genomes are present in the MGnify Genomes (Gurbich et al., 2023) Non-model Fish Gut v2.0 catalogue https://www.ebi.ac.uk/metagenomics/genome-catalogues/non-model-fish-gut-v2-0
