# Supplementary material for: A bacterial and viral genome catalogue from Atlantic salmon highlights diverse gut microbiome compositions at pre- and post-smolt life stages
Source: Anim Microbiome. 2025 Aug 11;7:85. doi: 10.1186/s42523-025-00453-5 (PMC12341145; doi:10.1186/s42523-025-00453-5)

# Supplementary Figures

##### **Supplementary Figure 1** - Comparison of completeness and contamination of genomes within species clusters comprising bins derived from single-assemblies (circles) and co-assemblies (square).
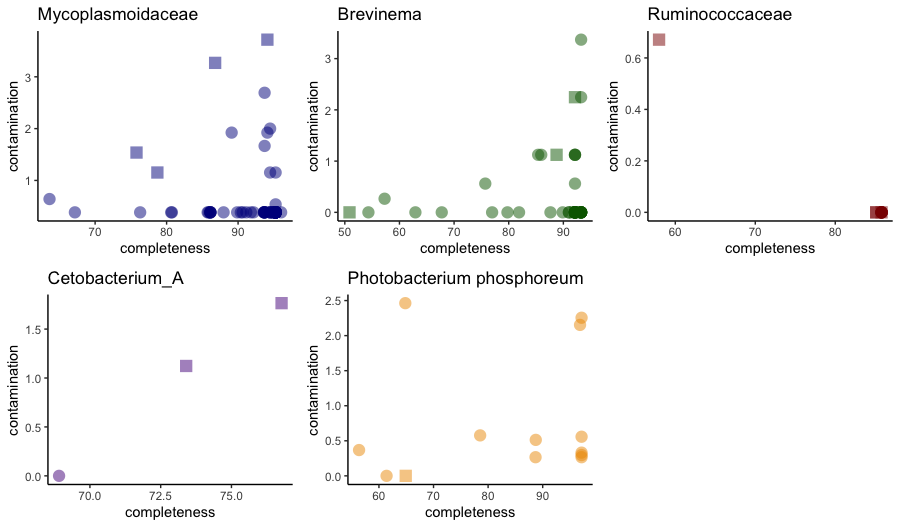


##### **Supplementary Figure 2.** Percentage of reads mapped per sample to the MAG catalogue of 11 bacterial species. Each point represents a sample, and the colour of the point indicates the number of species mapped.
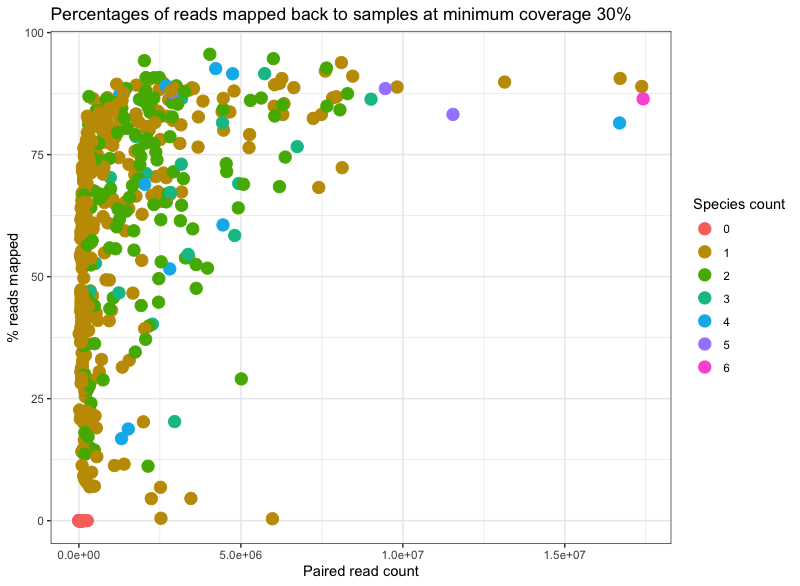

Supplement: Supplementary file 3 — Additional file 3: Genome mapping and quality figures. [file 42523_2025_453_MOESM3_ESM.docx]
