# Supplementary material for: A bacterial and viral genome catalogue from Atlantic salmon highlights diverse gut microbiome compositions at pre- and post-smolt life stages
Source: Anim Microbiome. 2025 Aug 11;7:85. doi: 10.1186/s42523-025-00453-5 (PMC12341145; doi:10.1186/s42523-025-00453-5)
Supplement: Supplementary file 4 — Additional file 4: Experimental designs. [file 42523_2025_453_MOESM4_ESM.pdf]

Experimental design and feeding trials

Metagenomic sequencing of Atlantic salmon gut samples was performed across three independent growth trials referred to as Trial A, B and C in the details below. All salmon were acclimated with a commercial feed prior to the trial for 2 weeks, followed by a trial period of ~2 months.

Trial A - fermented seaweed

The effect of seaweed on the salmon microbiome remains unknown, thus the rationale for including this feeding trial. Seaweed is a natural source of bioactive compounds with its own microbial community which can be further diversified after fermentation. Seaweed (sugar kelp, *Saccharina latissima*) was anaerobically fermented with an inoculum to add *Lactobacillus* to the feed. The feed was included in incremental doses from 0.0% to 2.0%. More details on this trial are described in Rasmussen et al. 2025. We sampled 180 adult salmon weighing between 300-600g and reared in seawater. The 180 salmon came from three experimental groups that were sampled at the start of the trial (n=60) as well as at the end of the trial for the control feed group with 0.0% seaweed inclusion (n=60) and the group receiving 2.0% seaweed inclusion (n=60).

Trial B - blue mussel meal

Traditional fishmeal consists of wild fish stocks which adds pressure to a fast growing sea farming industry and needs to be transported. Blue mussel by contrast can be grown locally and offer a more sustainable alternative. Spray-dried de-shelled blue mussels were used as a high quality blue mussel meal replacement for traditional fish meal. The blue mussel meal was included in incremental doses from 0.0% to 13.1% in the feed. At the end of the trial we sampled 180 adult salmon weighing between 200-400g and reared in seawater following the design described in Trial A. One group was sampled at the start of the trial (n=60) as well as at the end of the trial for the control feed group with 0.0% blue mussel meal inclusion (n=60) and the group receiving 13.1% blue mussel meal inclusion (n=60).

Trial C - blue mussel ensilage

This trial considered alternative processing of blue mussel meal to promote preservation of small peptides for better utilisation of the blue mussel proteins. Instead of a dry meal the blue mussel meal was stabilised using formic acid and formed into pellets. From this trial we sampled 180 juvenile salmon weighing between 10-90g and reared in freshwater to mimic the natural environment of juvenile salmon at this life stage.
